# Bioprinting Perfusion-Enabled Liver Equivalents for Advanced Organ-on-a-Chip Applications

**DOI:** 10.3390/genes9040176

**Published:** 2018-03-22

**Authors:** Tobias Grix, Alicia Ruppelt, Alexander Thomas, Anna-Klara Amler, Benjamin P. Noichl, Roland Lauster, Lutz Kloke

**Affiliations:** 1 Cellbricks GmbH, 13355 Berlin, Germany; at@cellbricks.com (A.T.); lk@cellbricks.com (L.K.); 2 Fachgebiet für Medizinische Biotechnologie, Technische Universität Berlin, 13355 Berlin, Germany; alicia.ruppelt@gmail.com (A.R.); aka@cellbricks.com (A.-K.A.); bn@cellbricks.com (B.P.N.); roland.lauster@tu-berlin.de (R.L.)

**Keywords:** bioprinting, stereolithography, liver equivalent, tissue engineering, bioink, 3D cell-culture, toxin testing, in vitro testing, drug development

## Abstract

Many tissue models have been developed to mimic liver-specific functions for metabolic and toxin conversion in in vitro assays. Most models represent a 2D environment rather than a complex 3D structure similar to native tissue. To overcome this issue, spheroid cultures have become the gold standard in tissue engineering. Unfortunately, spheroids are limited in size due to diffusion barriers in their dense structures, limiting nutrient and oxygen supply. Recent developments in bioprinting techniques have enabled us to engineer complex 3D structures with perfusion-enabled channel systems to ensure nutritional supply within larger, densely-populated tissue models. In this study, we present a proof-of-concept for the feasibility of bioprinting a liver organoid by combining HepaRG and human stellate cells in a stereolithographic printing approach, and show basic characterization under static cultivation conditions. Using standard tissue engineering analytics, such as immunohistology and qPCR, we found higher albumin and cytochrome P_450_ 3A4 (CYP3A4) expression in bioprinted liver tissues compared to monolayer controls over a two-week cultivation period. In addition, the expression of tight junctions, liver-specific bile transporter multidrug resistance-associated protein 2 (MRP2), and overall metabolism (glucose, lactate, lactate dehydrogenase (LDH)) were found to be stable. Furthermore, we provide evidence for the perfusability of the organoids’ intrinsic channel system. These results motivate new approaches and further development in liver tissue engineering for advanced organ-on-a-chip applications and pharmaceutical developments.

## 1. Introduction

Engineering tissues for in vitro organ models has always been a challenge. The creation of complex 3D tissues is motivated by the fact that 3D cell cultures commonly result in biology closely representing native tissues [1]. Nevertheless, complex tissues require cultivation conditions of elevated complexity, such as perfusion of the organoid in bioreactors like multi-organ-chips [2]. In order to empower their full potential, organoids can, not only be cultivated in monocultures resembling specific cell niches [3], but can also be combined in multi-organ cultures for metabolic and systemic studies [4]. Recent advances in bioprinting technology have enabled us to push the development of complex 3D tissues for in vitro applications even further, by setting up microfluidic channels within the printed organ equivalents for perfusion and the possibility of vascularization.

There are many different bioprinting techniques that have introduced additive manufacturing technology to the field of tissue engineering [5]. Among laser-induced-forward-transfer (LIFT) [6] and extrusion-based bioprinting [7], stereolithographic bioprinting combines many advantages and features the preparation of high-resolution hydrogels [8] with detailed architectures and embedded vascularization, supplying cell-laden tissues for long-term cultivation [9,10,11,12]. Current research pushes the trends and limitations of bioprinting technology [13], implementing solid-freeform manufacturing, formerly referred to as a tool for rapid-prototyping [14,15], into cell culture laboratories for tissue manufacture [16,17]. For the successful printing of tissue models, not only progress in 3D printing technology, but also the development of suitable bioinks, are necessary. Gelatin-based bioinks show great promise for embedding cells and creating suitable conditions for the proliferation and differentiation of many cell types [18,19,20]. By customizing bioink properties, specific cell–biomatrix interactions, such as cell migration [21] or directional matrix degradation [22], are controllable. 

In view of these developments, many tissue models have already been engineered for in vitro cell culture applications [23,24]. Ma et al. showed higher liver-specific gene expression levels and increased metabolic product secretion in a 3D bioprinted liver model consisting of human induced pluripotent stem cell (hiPSC)-derived hepatic progenitor cells, human umbilical vein endothelial cells, and adipose-derived stem cells. Furthermore, their model found both phenotypic and functional enhancements in comparison to 2D monolayer culture [25]. Tsang et al. reported the fabrication of 3D hepatic tissues by additive photopatterning of modified polyethylenglycol (PEG) hydrogels, whereas bulk and patterned hydrogels were compared, showing favorable performance in a continuous flow bioreactor over a 12 day cultivation period [26]. In addition, Lewis et al. reported architecture-dependent hepatic quality in a 3D printed gelatin scaffold. They found increased albumin secretion, CYP activity (CYP3A4 and CYP2C9), and bile transport in interconnected scaffolds compared to different geometries and 2D controls [27]. Other liver tissue models involve polyelectrolyte multilayer templates to combine primary hepatocytes and fibroblasts in a patterned co-culture. This approach results in controllable cell–cell and cell–surface interactions, as reported by Kidambi et al. [28]. In a similar approach, Puttaswamy et al. arranged HepG2 cells in a hexagonal liver lobule like structure, in which cells remained viable despite the application of an electric field for cell manipulation and positioning [29]. Using an enhanced field-induced dielectrophoresis trap, Ho et al. combined hepatic and endothelial cells, mimicking the morphology of liver lobule tissue with about 95% cell viability. Furthermore, an 80% enhancement of CYP1A1 activity was reported compared to non-patterned pure HepG2 cells [30]. Khetani et al. showed that tissue function depends on hierarchical structures extending from single cells to functional subunits that coordinate organ functions. In their multiwell culture system for human liver cells with optimized microscale architectures, phenotypic functions were maintained for several weeks. Their results emphasized the combination of different technologies to advance tissue engineering for ‘human-on-a-chip’ applications [31].

Here, we present a bioprinted tissue equivalent, representing the smallest functional unit of the liver. The printed lobule consists of a hexagonal structure possessing twelve channels running from the model edges to the central port. Channels are open at both sides, so that fluids can perfuse the complete construct.

In order to provide basic characterization of the printed liver equivalents, we chose standard tissue engineering analyses, including quantitative PCR, immunohistochemistry (IHC) staining and metabolic activity assays. Different protein targets were defined to classify the quality of the printed tissue compared to HepaRG cells cultivated in monolayer. We focused on albumin, cytochrome P450 3A4 (CYP3A4), cytokeratin 8/18 (Ck8/18), vimentin, multidrug resistance-associated protein 2 (MRP2) and zonula occludens-1 (ZO-1) expression. Albumin indicates the maturity of hepatocytes, whereby it is crucial to verify the presence of the protein using IHC [32]. Cytokeratin 8/18 is expressed by hepatocytes with proliferative capacity, which contribute to the tissues’ regenerative potential [33]. For toxicity screenings, the activity of the Cytochrome P450 enzymes is fundamental. Since CYP3A4 is one of the most important enzymes of that family, analyses were focused on its expression in order to provide a proof-of-concept for cytochrome activity in the printed liver equivalent [34,35]. One of the liver’s main functions is the conversion and transportation of substances. Thereby, the liver secretes bile, which is transported inter- and intracellularly by specific transporters. We, therefore, also investigated the expression of the MRP2, actively controlling bile in- and out-fluxes [36]. Functional hepatocytes require close cell–cell interactions through tight junctions, which are represented by peripheral membrane proteins, such as ZO-1 [37]. As the printed liver equivalents, not only contain HepaRGs, but also stellate cells, we chose vimentin, a protein of mesenchymal origin [38], to visualize cell distribution within the tissue.

## 2. Materials and Methods

### 2.1. Cell Culture

Cell culture components were purchased from Corning and cultures were incubated in HepaRG medium at 37 °C and 5% CO_2_, unless otherwise stated. HepaRG cells were obtained from Biopredic International (Rennes, France) and maintained as described by Gripon et al. [39]. Briefly, cells were cultured in HepaRG medium, consisting of William’s Medium E supplemented with 10% (*v*/*v*) fetal bovine serum (FBS), 100 units mL^−1^ penicillin, 100 µg mL^−1^ streptomycin, 5 µg mL^−1^ human insulin, 2 mM l-glutamine, and 5 × 10^−5^ M hydrocortisone hemisuccinate (Sigma-Aldrich, St. Louis, MO, USA). Undifferentiated cells were maintained in 75 cm^2^ tissue culture flasks (Greiner Bio One, Solingen, Germany) at a seeding density of 2 × 10^4^ cells cm^−2^ for two weeks. Induction of differentiation was initiated by allowing the cells to reach confluence by maintaining the cells in a growth medium for two weeks. Differentiation medium containing 2% (*v*/*v*) dimethyl sulfoxide (DMSO; Carl Roth GmbH, Karlsruhe, Germany) was added for another two weeks. Human hepatic stellate cells (SteCs) and their culture supplements were purchased from ScienCell Research Laboratories (Carlsbad, CA, USA). The cells were seeded at 5 × 10^3^ cells cm^−2^ in 75 cm^2^ tissue culture flasks in stellate cell medium for maintenance, according to the manufacturer’s instructions. Medium was exchanged every three days. Cells were harvested for further use at 90% confluence.

### 2.2. Bioink Preparation

For stereolithographic printing, two bioinks based on gelatin and PEG were used. Both bioinks were synthesized, as previously shown [40,41]. In short, 10 wt % gelatin (porcine skin Type B, Sigma) was dissolved in phosphate buffered saline (PBS) at 50 °C. After, 20-fold molar excess methacrylic anhydride (Sigma) was added and the reaction continued for 3 h. After reaction, the product (GelMA) was dialyzed against distilled water. Products were freeze dried and lyophilized for precise bioink preparation. Degradable PEG-bis-(acryloyloxy acetate) was synthesized in a two-step reaction. First, PEG-bis-chloroacetate was synthesized by reacting PEG (Sigma) with chloroaceryl chloride (Sigma). In the second step, acrylic groups were added by reacting the product with sodium acrylate (Sigma). Products were recovered by precipitation in cold ethylether (Sigma), dialyzed against distilled water and freeze-dried for long-term storage. The photoinitiator lithium phenyl-2,4,6-trimethylbenzoy phosphinate was used at 0.1 wt % in all bioinks. For the bioprinting process, cells were mixed with bioink-solutions containing the photoinitiator to form a bioink cell suspension ready for photopolymerization. 

### 2.3. Tissue Model and Printing Process

**Figure 1:**
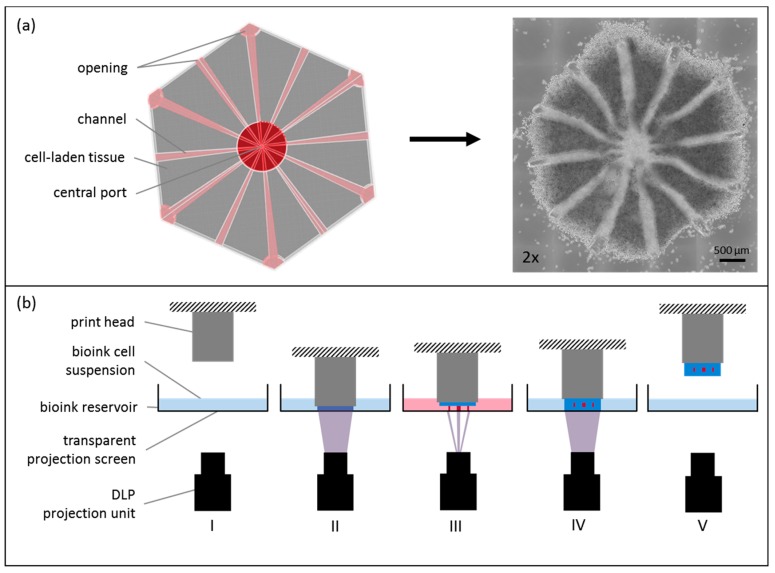
Cellbricks printing process of liver model. (**a**) During the printing process, the virtual 3D model (top view) is translated into a cell-laden multi-material hydrogel; here shown in a 2× microscopic image; (**b**) The printing process is visualized schematically. (I) The bioink-cell suspension is filled in the printers bioink-reservoir. (II) Digital-light-processing (DLP) projection is done layer-by-layer through the transparent reservoir bottom onto the print head. (III) After each layer, the print head moves upwards for the next layer. (IV) Each layer was photopolymerized onto the previous layer. (V) After complete printing, the formed tissue model is removed from the print head, ready for cultivation.

The liver equivalents are designed with hollow channels to allow for perfusion of the organoid. Within the hexagonal construct, there were twelve channels running from the model edges to a central port. The channels were open at both sides (Figure 1a). The printed liver model consisted of two materials. Channels were printed with degradable PEG at 7 wt %. For the cell-containing structures (Figure 1a, in grey), HepaRGs were harvested, mixed with SteCs 24:1 and resuspended in GelMA, thereby forming the main bioink at 7% (*w*/*v*), possessing a cell density of 10 × 10^7^ cells/mL. A model mimicking the sinusoidal structure of the liver lobule was designed with a diameter of 4 mm, using computer-aided design (CAD) software (Rhinoceros 5, McNeel Europe, Barcelona, Spain). The CAD file was processed using a Cellbricks Bioprinter and was printed, layer-by-layer. as shown in Figure 1b. During printing, the bioink was changed automatically according to the material and cells used for the current structure.

During the printing process, each layer of the tissue construct was photopolymerized directly onto the print head using blue light illumination for 30 s per layer. Stereolithographic printing technology enabled us to manufacture six tissue models, in parallel, during one printing process. Each model contained a total of 10^6^ cells. After printing, the constructs were detached from the bioprinter and placed in a 24-well plate filled with 1 mL cell culture medium for cultivation. Tissue constructs were incubated at 37 °C and 5% CO_2_ over 14 days of cultivation time. Two time points, directly after printing and 14 days after printing, were chosen for analysis. Medium changes were performed every day.

#### 2.4. qPCR

Gene expression of albumin, CYP3A4, ZO-1 and MRP2 were analyzed by qPCR, which was performed directly after printing, on day zero, as a control, and after 14 days of cultivation. RNA isolation was performed using the NucleoSpin^®^ RNA isolation Kit (Macherey-Nagel, Düren, Germany) by harvesting the tissue model in RA1 buffer containing 1% β-mercaptoethanol according to the manufacturer’s protocols. A total of 150 ng of RNA was reverse transcribed into cDNA with the TaqMan^®^ kit (Applied Biosystem, Foster City, CA, USA), for each sample. Quantitative RT-PCR experiments were conducted according to the manufacturer’s protocols using the Stratagene system (Agilent Technologies, Waldbronn, Germany) with a SensiFast SYBR No-ROX One-Step kit (Bioline, Luckenwalde, Germany). Used primers and their sequences are presented in Table 1. Cycle threshold and melting curves were determined using LightCycler software and results were processed using the 2-ΔΔCt method for relative gene expression analysis [42,43]. Changes in gene expression were normalized using the TATA-Box binding protein (*TBP*) as a housekeeping gene. For each time point, six tissue samples were taken. Monolayer cultures were measured with *n* = 10. Statistical analyses, such as the unpaired *t*-test, were performed in Prism 7 (Graphpad Software, La Jolla, CA, USA).

### 2.5. Immunohistochemistry 

For immunohistological analysis, constructs were embedded in Tissue-Tek^®^ (Sakura, The Netherlands), frozen in liquid nitrogen and cryosectioned at a thickness of 10 µm. Sections were fixed in acetone at −20 °C for 10 min. After washing in PBS and blocking in 10% goat serum for 20 min, immunostaining was performed using primary antibodies (ThermoFisher, Waltham, MA, USA) targeting albumin, CYP3A4, ZO-1, MRP2, vimentin and cytokeratin 8/18 at 4 °C overnight. Secondary antibodies (goat-anti-mouse and goat-anti-rabbit CF594, Biotium, Fremont, CA, USA) were incubated for 45 min at room temperature with 1:5000 DAPI for cell nuclei staining. Afterwards, coverslips with mounting solution were added to seal the staining. Fluorescent microscopy was performed using a Biorevo BZ-9000 (Keyence, Osaka, Japan). Viability was determined using TUNEL/Ki67 double staining, using the Apo-Direct Apoptosis Detection Kit (eBioscience, San Diego, CA, USA), according to the manufacturer’s protocols, in combination with Ki67 antibody (eBioscience, San Diego, CA, USA).

### 2.6. Metabolic Analysis

Daily medium samples were taken for the determination of glucose-, lactate- and lactate dehydrogenase (LDH)-content. Absorbance-related measurements were performed in 384-well plates (Greiner Bio-One, Solingen, Germany) in a microplate-reader (FLUOstar Omega, BMG Labtech, Ortenberg, Germany). LDH activity in the medium was measured using a Cytotoxicity Detection Kit PLUS (Roche Diagnostics GmbH, Mannheim, Germany) according to the manufacturer’s protocols. The average absorbance per minute (ΔA/min), at 450 nm, was determined over three minutes using medium as a reference. As a positive control, samples were treated with 0.1% Triton X-100 for two hours and supernatants were analyzed. Daily glucose consumption was measured with the Glucose Kit Glu 142 (Diaglobal, Berlin, Germany) according to manufacturer’s protocols, using medium as standard. Lactate concentration was screened in daily medium supernatants using a Lactate Kit Lac 142 (Diaglobal), according to the manufacturer’s protocols. Absorbance was measured at 520 nm using standards (10 mM/mL) as a reference.

## 3. Results

### 3.1. Morphological Analysis after Printing

**Figure 2:**
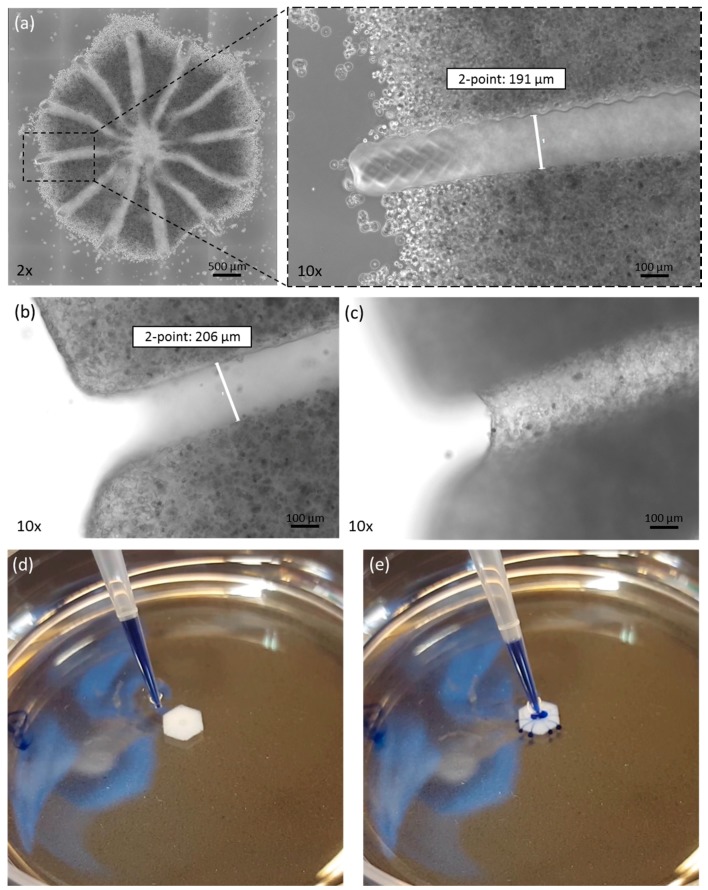
Morphology of the printed tissue model. (**a**) Microscopic picture of liver model directly after printing. At 10× magnification, the channel-structure is clearly visible. After complete dissolution, the channel is a hollow structure, as seen by focusing on (**b**) the edges or (**c**) the channel bottom. The channel structure is hollow, as demonstrated by flushing with trypan blue dye (**d**,**e**).

The printed liver equivalents had a diameter of 4 mm from edge to edge. This size was verified in all printed constructs. A mean of 4053.6 µm with a standard deviation of 67.2 µm was determined (based on 30 samples). Multiple tissue constructs printed at the same time displayed high equality in morphology and cell distribution. Cells were found within the printed matrix at a high density and with a homogenous distribution (Figure 2a). The channel–structure was defined by the sacrificial matrix, as shown in Figure 2a. The channels had a diameter of approx. 200 µm, with a standard deviation of under 10 µm, as shown in Figure 2a,b. Immediately after printing, some cells were loosely attached to the model edges. After washing, these cells were removed and only cells that were truly incorporated within the bioink remained. After three days in culture, the PEG was fully degraded, as seen in Figure 2, by focusing on the edges (Figure 2b) and the bottom (Figure 2c) of the channel. Furthermore, we were able to flush the complete channel system, thus demonstrating the organoids’ suitability for cultivation under perfusion (Figure 2d,e and Supplementary Video S1: Perfusion of 3D bioprinted liver equivalent.).

### 3.2. Viability after Bioprinting

**Figure 3:**
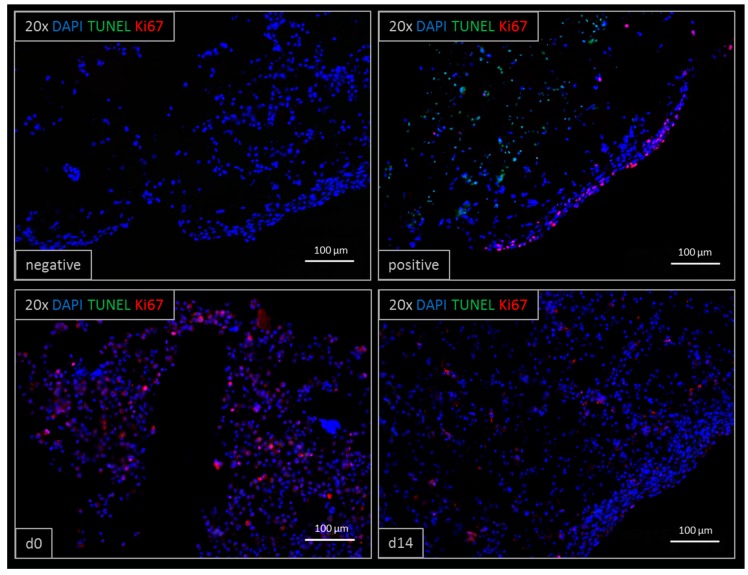
TUNEL/Ki67 staining of printed liver constructs. Proliferative cells (Ki67 positive) are visible both at day zero and after 14 days of cultivation. Ki67 positive cells appear less at day 14 compared to day 0. Apoptotic cells (TUNEL positive) were observed in the positive DNase-treated control. The negative control did not show any unspecific staining.

Viability was confirmed by TUNEL/Ki67 staining (Figure 3). Printed liver equivalents showed high viability throughout the cultivation time of 14 days. Ki67-positive, proliferative cells were observed at day zero and day 14, whereas TUNEL-positive, apoptotic cells could only be observed in the positive control treated with Triton X-100. Less proliferative cells were observed at day 14 compared to day zero, right after printing.

### 3.3. Glucose, Lactate and LDH Metabolics

**Figure 4:**
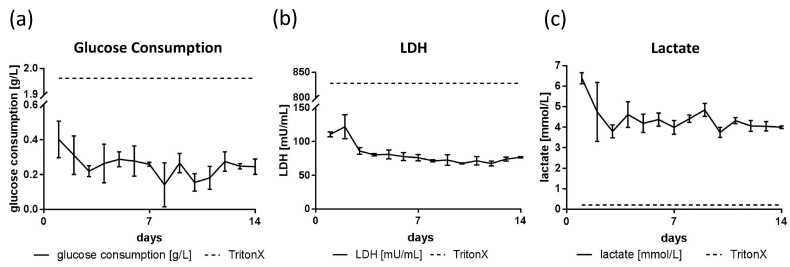
Metabolic data of glucose consumption, lactate dehydrogenase (LDH) and lactate concentration over 14 days of cultivation. (**a**) Average glucose consumption ranged from 0.14 to 0.4 g/L. Cells treated with Triton X-100 consumed more than 1.95 g/L. (**b**) The LDH release started at about 100 mU/mL, but stabilized at around 70 mU/mL three days post-printing. Triton X-100 treated cells released 830 mU/mL in average. (**c**) Lactate concentration was measured between 3.7 and 6.4 mmol/mL. Cells treated with Triton X-100 showed 0.2 mmol/mL lactate concentration in the culture medium.

Glucose consumption was observed to be higher at the beginning of cultivation, measuring around 0.15 mg to 0.3 mg glucose consumption per day (Figure 4a). Complete medium changes with a maximal glucose concentration of 2 mg per culture were performed every day. Cells treated with Triton X-100 consumed more than 1.95 g/L glucose per day. LDH levels were observed to be far below levels of the positive control, indicating viable cells throughout the 14 day of cultivation. LDH levels started at about 100 mU/mL in the first two days of cultivation, but declined and stabilized around 70 mU/mL in the following days (Figure 4). Tissue constructs treated with Triton X-100 represented the positive control for apoptotic cells. In these samples, LDH levels rose to 830 mU/mL. Lactate content was found to be between 3.7 and 6.4 mmol/mL. Similar to observations made for LDH, these levels were elevated in the first two days of cultivation, dropping down to an average lactate concentration of 4.5 mmol/L (Figure 4). Lactate content in pure culture medium without cells was found to be 3 mmol/L (data not shown).

### 3.4. qPCR Marker Expression

**Figure 5:**
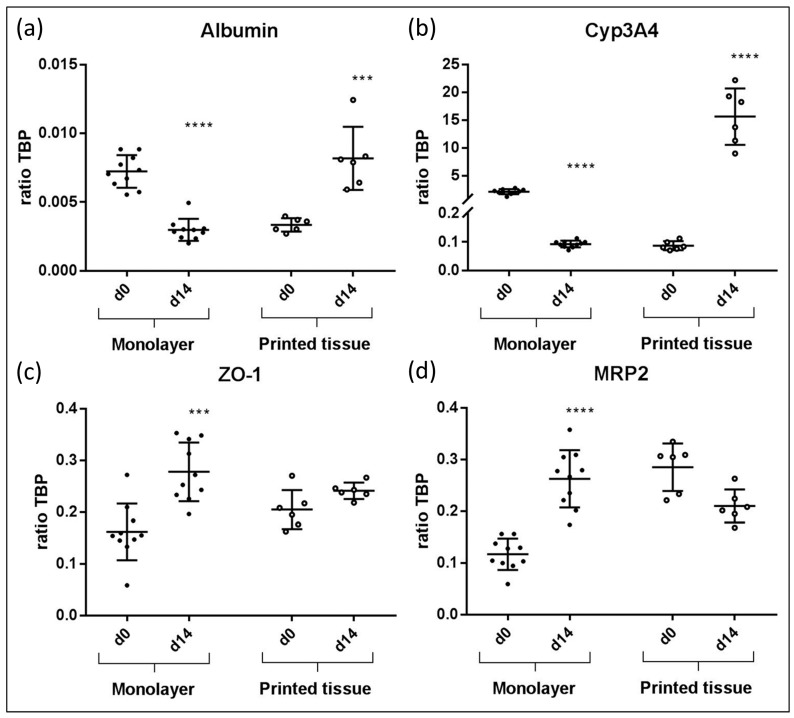
Gene expression results from bioprinted liver tissue compared to HepaRG monolayer cultures. In each graph, the qPCR results of albumin (**a**), CYP3A4 (**b**), ZO-1 (**c**) and MRP2 (**d**) of monolayer cultures and printed liver constructs at day 14 are compared to day 0, respectively. Results showed statistically significant differences. (*** *p* < 0.0005; **** *p* < 0.0001).

The following gene expression results for 3D printed liver equivalents and for HepaRG monolayer cultures are summarized in Figure 5. In printed liver equivalents, albumin expression was found to rise significantly from day zero to day 14 (Figure 5a). Monolayers showed the opposite effect; a significant drop in albumin expression from day zero to day 14 was detected. Overall, albumin expression levels were higher in 3D bioprinted liver equivalents than in HepaRG monolayers. Similar results were observed in terms of CYP3A4 expression. Gene expression in the bioprinted tissue constructs appeared to be, on average, 150-fold higher on day 14 compared to day zero. Monolayers showed a significant drop in CYP3A4 expression from day zero, right after maturation, to day 14, two weeks in culture after maturation. Overall CYP3A4 expression appeared to be more than seven times higher in 3D bioprinted tissues compared to monolayer cultures at their highest expression levels (monolayer: Day zero; prints: Day 14) (Figure 5b). The tight junction protein ZO-1 was expressed in both 3D tissues and monolayer cultures throughout the experiments with a significant increase of expression in monolayers. Overall expression levels appeared to be similar, but were slightly higher in monolayers than they were in the printed liver equivalents at day 14 (Figure 5c). MRP2 expression dropped slightly in bioprinted liver equivalents and expression remained stable over the two week cultivation. In contrast, the monolayer demonstrated a significant increase in MRP2 expression levels from day 0 to day 14 (Figure 5d).

### 3.5. Immunohistochemistry

**Figure 6:**
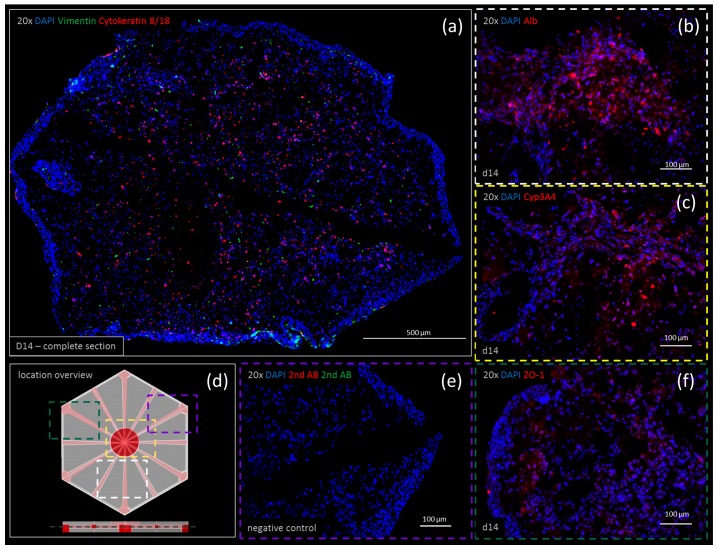
Immunohistochemistry staining of printed liver constructs showing expression patterns of vimentin (green) co-stained with cytokeratin 8/18 (red) after 14 days of cultivation shown in a complete section of the print (20× merged) (**a**). The expression of (**b**) albumin (Alb), (**c**) CYP3A4 and (**f**) ZO-1 is shown in a single staining after two weeks of cultivation. The location overview (**d**) shows where each staining is located within the construct. The negative control (**e**) verifies the results.

Negative controls for both the double and single staining showed no unspecific antibody interactions (Figure 6e). Immunohistochemistry is shown at day 14 for vimentin, cytokeratin 8/18, albumin (Alb), CYP3A4 and ZO-1. Vimentin and cytokeratin 8/18 were co-stained visualizing the cell distribution. Stellate cells visualized by positive staining of vimentin (Figure 6a, in green) were found to be distributed homogenously throughout the printed tissue. Some accumulation due to cell proliferation was observed at the bottom edge. Cytokeratin 8/18 was predominantly stained within the center of the organoid. Only a few cytokeratin 8/18-positive cells were found on the edges, within a dense cell layer surrounding the whole organoid. Both albumin and CYP3A4 appeared to be highly expressed (Figure 6, albumin (b) and CYP3A4 (c)). Their expressions were found to be stronger at day 14 compared to day zero (data not shown). ZO-1 expression was observed in densely-populated areas throughout the printed tissue (Figure 6f).

## 4. Discussion

In this study, we successfully demonstrated the stereolithographic printing principle of a complex hepatic tissue construct with an intrinsic hollow channel system. The results build a foundation for future detailed characterization and development of a functional liver model with possible applications in metabolic and toxicologic assays. The translation of a 3D digital drawing into a highly-structured, cell-laden hydrogel-based organoid was successfully performed (Figure 2). Right after printing, some cells were loosely attached to the printed model due to the high cell density within the bioinks. These cells can easily be washed away, and only the designed structure with the incorporated cells remains. Multiple tissue constructs, printed at the same time, showed a high equality in morphology and cell distribution constituting an accurate representation of the intended design for the tissue model, thus reproducible printing of organoids was demonstrated. Furthermore, apoptotic cells could only be found in the positive control after two weeks of cultivation (Figure 3). Proliferating cells were found both at day zero and after two weeks in culture demonstrating a high viability for cultivation over at least 14 days. More Ki67 positive cells were observed at day 0 compared to day 14, which indicates a loss of proliferative capacity due to cell differentiation. In this study, we compared HepaRG cells cultivated in monolayer with the same cells incorporated in the printed lobular constructs. For both cultivation methods, cells were matured over two weeks in monolayer. Afterwards one half of the cell culture was printed and cultivated in 3D, the other half remained in the monolayer culture. Unfortunately, we had to exclude stellate cells from the monolayer controls, as these cultures appeared unstable over 14-day cultivation, contracting into an elliptic random construct, whereas monolayers without stellate cells remained stable over the two week cultivation period. Both monolayer and printed tissue cultivations were performed over 14 days. Overall gene expression of selected markers was found to be higher in the printed tissues compared to the monolayer cultures, confirming the direct effect of the three-dimensional cultivation on the cells’ biology (Figure 5). In the printed liver equivalents, the tight junction protein ZO-1 was found to be stably expressed over 14 days of cultivation. At day 14, its standard deviation dropped to a minimum. As cells expressed tight junctions when in contact with each other, and more organoids showed a higher ZO-1 expression at day 14 compared to day 0, these results suggest some cell proliferation within the hydrogel. One of the models already showed maximal expression on day zero, maintaining this level over the entire cultivation time. Since the cells are pre-differentiated over 14 days in monolayers, detached, suspended in the bioink and then printed in high cell density, tight junction protein expression might remain high from the first day on, but this assumption requires further experiments. MRP2 expression was found to increase in monolayers, whereas there was a slight decrease in expression levels found in printed tissues (Figure 5). In monolayer cultures, the HepaRGs proliferate until complete confluency, differentiating and forming bile ducts with transporter expression. In the printed tissues, cells are incorporated in a hydrogel at a given concentration. Thus, the cells are surrounded by the printed matrix, but not all cells interact with each other. Cells that expressed MRP2 in the confluent monolayer prior to printing might not have any cell–cell contact after printing within the gelatin hydrogel. This might explain the decrease in MRP2 expression in our printed tissues over 14 days of cultivation. An increase in bioink cell concentration in future organoids might support the expression of MRP2 as more cells stay in contact with each other, supporting the formation of bile ducts and the main bile acid transporters [36]. There was a high difference in protein expression at day zero, comparing printed tissue and monolayers, although the cells were pre-differentiated equally in both experiments. Albumin expression was found to be two-fold higher and CYP3A4 expression was found to be even 20-fold higher in monolayers, than in printed tissues, at day zero (Figure 5). This difference can be explained by the procedure used to take the RNA samples, as different cell handling results in metabolic alterations [44]. For the monolayers, RNA samples were taken by lysing the attached cells directly from the tissue culture flask so that no changes in expression were expected. For printed tissues, cells were detached, mixed with bioink, resulting in a single-cell-suspension, and printed within the hydrogels. Right after the printing process, the tissue constructs were lysed to extract the RNA. The cells remained in suspension before the bioprinting process, thus downregulating liver-specific gene expression (albumin, CYP3A4, MRP2). Only ZO-1 was not affected by this phenomenon in all printed tissues. Nevertheless, this procedure was chosen to investigate the actual changes in gene expression from day zero to day 14. Monolayer controls lose albumin and CYP3A4 expression over the two weeks cultivation period. Usually, HepaRG functionality in monolayers is maintained by adding DMSO after the pre-differentiation. As the printed tissues are cultivated without DMSO, monolayer cultures were also kept without DMSO, resulting in a loss of hepatic function. The printed tissues, however, maintained hepatic functionality under these native conditions.

Protein expression was verified by immunohistology, being in accordance with the results from the qPCR experiments (Figure 6). Some morphological deformations were observed in cryosections due to the freezing and cutting procedure of the tissues. As some sections appeared to be squeezed in one direction (e.g., y-direction, Figure 6a) these deformations were most likely introduced by the cutting procedure. In Figure 6a,e, a part of the channel structure is visible (black linear area within the staining). Not all channels are visible at once due to the cutting angle. An accumulation of vimentin positive cells (stellate cells) was observed at the bottom edge of the tissue section (Figure 6a). As HepaRGs and SteCs are homogenously mixed in the bioinks, and SteCs were found homogenously distributed throughout the printed constructs at day zero, this accumulation suggests the proliferation or migration of stellate cells at this spot. As cells at the edges of the printed tissues might not be fully incorporated in the printed matrix, they have more space to proliferate and populate the construct surface. Cytokeratin 8/18-positive cells within the same staining suggest some proliferative hepatocytes, representing an active tissue with regenerative capabilities.

Metabolic analyses revealed the production of small amounts of lactate (Figure 4c) in accordance with glucose consumption (Figure 4a). These results suggest oxygen limitation so that glucose is converted to lactate. The oxygen limitation might be a result of the medium amount used for cultivation. In this study, the printed tissues were cultivated in a 24-well format with 1 mL of medium. Manufacturers like Greiner Bio-one suggest using only 0.5 mL of medium in this format. The higher liquid level limits the amount of oxygen at the bottom of the culture well, where the organoid can be found [45]. In future experiments, medium levels will need to be adapted and cultivation under perfusion will help to alleviate this problem. As LDH levels appeared to stay stable after the first two days (Figure 4, center), the printed tissues stay viable. Metabolic data suggest homeostasis, as no significant increases or drops in neither glucose consumption nor lactate concentration were visible.

In future experiments, the printed liver organoid will be cultivated in a multi-organ-chip platform, which facilitates the in- and efflux of oxygen, carbon dioxide, nutrients and metabolites [46]. Co-cultivation with other organ models, such as pancreatic islets, neural tissue or skin, might pose a promising strategy in testing and optimizing the printed liver equivalents at their current state [4,47,48]. As the channel system allows for perfusion (Figure 2d,e and the supplementary video), adding endothelial cells to form tight homogenous channel walls is a crucial extension to the development of this organ model for physiologic perfusion experiments. In native tissue, substances need to pass endothelial barriers before reaching biological active hepatocytes [49], thus, the addition of endothelial cells will support hepatic polarization, leading to a higher biological activity and a more detailed physiology [50,51].

## 5. Conclusions

In this study, a complex liver organoid was precisely printed using a stereolithographic bioprinting approach. We were able to print a hollow channel system within the cell-laden hydrogel. The printed liver tissue equivalents were found to have higher albumin and CYP3A4 expression over a two week cultivation period, when compared to monolayer controls. Tight junction protein ZO-1 and MRP2 expression remained stable in the printed tissue. However, monolayer controls showed an increase in the expression of these genes, so there still is potential to adapt cell densities within the printed organoids. In its current state, we found that the printed liver organoid has great potential for future lab-on-a-chip and organ-on-a-chip applications, as medium can flow through the channel system within the tissue model, preparing it for cultivation under perfusion. We successfully established the stereolithographic printing technology, thus enabling development of the model. Now that we demonstrated the feasibility of the printing principle, detailed analyses of the major enzymes of the cytochrome P450 family are crucial to fully characterize the model for applications in metabolic and toxicology assays. Furthermore, the application of potential toxic substances will give insight into the enzyme kinetics and overall organoid performance. As our printing technology is constantly being developed, we aim to incorporate endothelialized channels in the future to support physiologic hepatic polarization for long-term toxicity screenings. The organoid was established using HepaRG cells, but, as the presented bioprinting technique it is not limited to cell lines, we suggest the integration of iPSCs or a primary material for potential personalized medicine applications.

## Figures and Tables

**Table 1:** Primer sequences used for qPCR.

Primer	Forward	Reverse
Albumin	TCAGCTCTGGAAGTCGATGAAAC	AGTTGCTCTTTTGTTGCCTTGG
CYP3A4	GGAAGTGGACCCAGAAACTGC	TTACGGTGCCATCCCTTGAC
ZO-1	TCTCGGAAAAGTGCCAGGAAG	CCCTCGGAAACCCATACCAG
MRP2	GGGGACACTGTTGGCTTTGTTC	CCCAGGGTGCCTCATTTTCCA
TBP	CCTTGTGCTCACCCACCAAC	TCGTCTTCCTGAATCCCTTTAGAATAG

CYP3A4: cytochrome P_450_ 3A4; ZO-1: zonula occludens-1; MRP2: multidrug resistance-associated protein 2; TBP: TATA-Box binding protein.

## Supplementary Materials

The following supplementary material is available online at http://www.mdpi.com/2073-4425/9/4/176/s1, Video S1: Perfusion of 3D bioprinted liver equivalent.
